# Quality of life (QOL) and depression in HTLV-1 carriers

**DOI:** 10.1186/1742-4690-8-S1-A65

**Published:** 2011-06-06

**Authors:** Ana V Galvão-Phileto, Ney Boa-Sorte, Ramon A Kruschewsky, Bernardo Galvão-Castro

**Affiliations:** 1 Centro de HTLV, Escola Bahiana de Medicina e Saúde Pública, Salvador, Bahia, Brasil

## Background

The prevalence of depression in HTLV-1 infected patients is about 30%. However, there are few studies about depression and QOL in patients infected with HTLV-1. The aim of this study is to verify the association between depression and predictors of QOL of people living with HTLV-1.

## Material and methods

A cross-sectional study was carried out from March to November, 2009 in Salvador, Brazil. The instruments used were a questionnaire for the collection of clinical and epidemiological data, the Mini International Neuropsychiatric Interview, Brazilian Version 5.0.0 (M.I.N.I.) to estimate the rate of depression and the short version of the WHO quality of life scale (WHOQOL-BREF).

## Results

The overall prevalence of depression was 34.7% (33out 95). Depression was significantly associated with poorer QOL in physical, psychological “social relationship” and environment domains.

## Conclusion

This study confirms the high prevalence of depression in individuals infected with HTLV-1. It also shows that depression significantly affected the QOL of these individuals. Since treating depression could improve the QOL, we strongly recommend early identification and referral of patients with depression. This measure should be included into intervention programs designed for individuals infected HTLV-1.

